# Implementation of pharmacist-managed early switch from intravenous to oral therapy using electronic identification at a tertiary academic hospital

**DOI:** 10.1016/j.jsps.2021.03.006

**Published:** 2021-03-23

**Authors:** Alaa Babonji, Bayan Darwesh, Maha Al-alwai

**Affiliations:** a Clinical Pharmacist at King Abdulaziz University Hospital, Jeddah 23226-3523, Saudi Arabia; b Director for the Pharmaceutical Services Department at King Abdulaziz University Hospital, Jeddah 23734-6850, Saudi Arabia^1^; c Medical Manager at Pfizer, Jeddah 23734-6850, Saudi Arabia^2^; d Medical Microbiology and Parasitology Department, Faculty of Medicine, King Abdulaziz University, Jeddah 21422-5107, Saudi Arabia; e Infection Control and Environmental Health, King Abdulaziz University Hospital, Jeddah 21422-5107, Saudi Arabia

**Keywords:** Intravenous, Oral, Switch, Pharmacist, Physician, Education, Protocol, IV, Intravenous, PO, Oral, PMES, Pharmacist managed early switch from IV-PO therapy, LOS, Length of hospital stay, NPO, Nil per os route, NGT, Nasogastric tube, DE, Direct Efficacy, OE, Overall Efficacy

## Abstract

Overutilization of intravenous (IV) medications can result in drug shortages, which is one of the major health care crisis, in addition to increasing costs, length of hospital stays (LOS) and the associated complications. We hypothesized that IV therapy was overused at our hospital where oral (PO) was applicable, and that the implementation of IV-PO protocol could result in a cost-effective practice. Hence, we aimed at assessing impact and outcomes of implementing such a protocol.

A single center, prospective quasi-interventional study conducted at tertiary academic hospital. A protocol was implemented targeting 17 medications, with educational sessions to medical staff during a 5-month phase. IV orders of 48 h or more, among adult patients at medical or surgical wards with no contraindication to PO route were eligible. Once eligible, pharmacists send interventions using hospital’s computerized order entry system, and physicians’ responses were monitored on daily basis. Efficacy was estimated by percentage of switch recommendations that resulted in effective switch to PO medication. Cost-minimization analysis was used for course cost between the control phase and intervention phase. Length of hospital stay (LOS), readmissions within 90 days and in-hospital mortality were analyzed as secondary outcomes.

During intervention phase, 781 patients had at least one IV order switched to PO. Gastric acid-reducing agents (GARAs) accounted for the most IV prescriptions (50.4%), followed by antibiotics (39.6%). Pharmacists carried out 2677 interventions to which switch recommendations were issued in 1185 (44.3%). Primary switch recommendations (N = 677) led to effective switch in 60.7% cases. These included per protocol switch (8.9%), switch to another PO (2.5%), spontaneous switch by physician (17.6%) and IV discontinuation (31.8%). The overall efficacy was estimated as 62.8%. The intervention was associated with reduced IV consumption from 4,574–18,597 vials in control phase to 3,654–15,546 vials in intervention phase, which resulted in overall cost saving of 50,960.8 SAR ($13,589.5), with an average monthly cost saving of 10,192.2 SAR ($2,717.9).

Pharmacist-managed early switch from IV-PO therapy, with physicians’ education, showed significant reduction in IV medication use in our hospital. By reducing unnecessary IV use, this strategy enabled considerable cost savings, besides the potential advantages of convenience and safety.

## 1 Introduction

The choice for route of administration for any drug is directed by the achievement of the optimal bioavailability for the desired therapeutic effect and good tolerance. Other factors which could affect choice of route of administration include drug availability, clinical status, and patient’s preference (Benjamin et al., 2012, Kuper, 2007, Zhou et al., 2015).

Intravenous (IV) route is the preferred dosage form in several clinical situations; notably, in acute and clinically unstable patients, or in patients with compromised oral absorption, and if no other dosage forms are available or comparable in efficacy. It is also the most common route in hospitalized patients, primarily in patients with prolonged hospital stay. Nevertheless, a plethora of evidence suggests that inappropriately used IV route is a reflection of poor quality of care, especially in inpatient settings (Buyle et al., 2012, van den Bosch et al., 2016).

Overuse of IV route exposes to specific safety concerns due to increased risk of medication errors during preparation and administration process (such as dose calculation, dilution, and injection). Such errors may be harmful to the patient and enfold a greater risk for serious adverse events compared to the oral dosage forms (Abbasinazari et al., 2013, Dart and Rumack, 2012, McDowell et al., 2010). On the other hand, overutilization of IV medication can result in drug shortages, which is one of the major healthcare challenges in the modern era, in addition to increasing costs, length of hospital stays (LOS) and the associated risk of IV line complications (Fox et al., 2014, Gray and Manasse, 2012, Stengård, 2014). Therefore, appropriate use and management of IV medication is essential for public health and patient’s safety.

One of the aspects of IV overutilization is the continuation of an IV prescription in a patient where oral formulations could be safely and effectively considered (Holloway and van Dijk, 2011, Mok et al., 2016). While IV-PO switch may admittedly be unsafe in some defined cases, an early switch may be recommended in several other cases. This was notably observed in antimicrobial prescribing and is often associated with lack of awareness among the physicians (Engel et al., 2013, Hammad et al., 2015, Lee et al., 2012, Shrayteh et al., 2014, Warburton et al., 2014).

Therefore, several hospital-based interventions have been described to be efficient in improving IV prescribing practice among physicians by promoting early switch from IV-PO based on defined criteria and following an established protocol. These interventions used various methods and achieved satisfactory results in terms of meeting safety. Besides, it also resulted to be cost effective due to significant decrease in IV drug consumption (Mertz et al., 2009, Stengård, 2014, van Niekerk et al., 2012, Vanstraelen et al., 2013). Most of these interventions were conducted by or involved clinical pharmacists in a stewardship program, among a multidisciplinary team, to enable successful implementation of the program.

We hypothesized that IV therapy was overused where PO was applicable at our hospital, and that the implementation of a pharmacist managed early switch from IV-PO therapy (PMES) could result in a cost effective practice. Hence, we aimed at assessing the efficacy in prompting early IV to PO medication switch of a PMES protocol combined with health care system enhancements and educational sessions to the multidisciplinary team in a tertiary academic hospital. We further evaluated the impact of the PMES on IV medication consumption, overall medication costs and patients’ safety.

## 2 Methods

### 2.1 Design & setting

This was a single center, open label, prospective quasi-interventional study that was conducted from November 2017 to August 2018. It’s a 1,067-bed tertiary academic hospital with approximately 43,950 admissions yearly. On an average, 340,695 IV preparations are prepared annually and pharmacist interventions for these preparations account for an average of 11,333 per year. The study protocol was approved by the Research Ethics Committee of the university, which waived the patient’s consent requirement.

### 2.2 Population

**Fig. 1:**
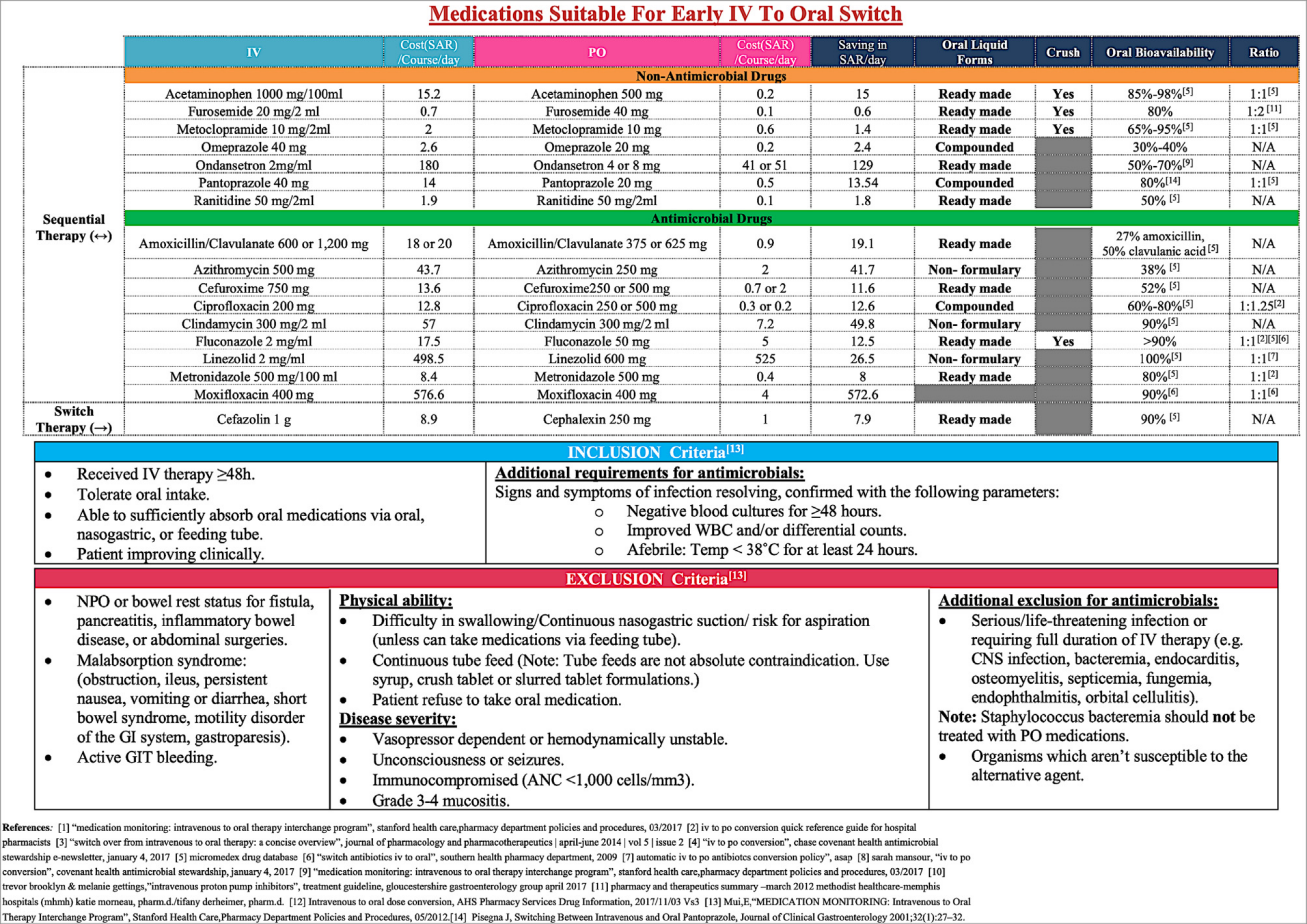
Hospital protocol for switching from IV-PO therapy.

Adult patients 18 years or older admitted at medical or surgical wards who were on targeted IV therapy for 48 h or more and who met hospital’s IV-PO inclusion criteria protocol. Patients who were on STAT orders, prophylactic orders, can’t tolerate oral route, active gastrointestinal bleeding, clinically unstable or had life-threatening infection requiring IV therapy were excluded (Fig. 1).

### 2.3 Intervention

The present PMES was conducted among hospitalized patients following 3 major steps, namely, system enhancements, protocol preparation, and implementation. A similar design has been implemented previously (van Niekerk et al., 2012).

#### 2.3.1 System enhancement

The hospital uses a health information system in which modifications are possible. Enhancements to the clinical pharmacist interface window were done to facilitate accessing patient’s information efficiently. These enhancements enabled generation of list of current inpatients at the hospital, with the ability to select units, patient’s age, diet, and a shortcut to access full patient’s profile. The list can be exported and saved as excel sheet to easily document and follow up the interventions on a daily basis.

#### 2.3.2 Protocol preparation

The hospital protocol for switch from IV-PO therapy was designed and approved by the Pharmacy and Therapeutics Committee and was discussed at the nursing-pharmacy committee. Prior to the current study, there was no related protocol in the hospital. A MEMO was sent to the involved departments. Inclusion and exclusion criteria were adopted from the Stanford Health Care protocol (Mui, 2013) including some adaptive modifications. A total of 17 targeted IV medications were included (Fig. 1), which had comparable bioavailability (Fischer et al., 2003) and are available as formulary in the hospital. Ten of the targeted medications were antimicrobial agents. A similar choice of targeted medications was adopted by Fischer et al. and Mok et al. (Fischer et al., 2003, Mok et al., 2016). The protocol was designed to display the targeted IV medications and their corresponding appropriate PO alternative therapy. In addition, it also highlights the cost of the average dose per day for IV and PO medications along with cost saving when choosing a PO agent for each IV drug. Additionally, whether PO medication can be crushed for tube feeding and the complete bioavailability of the targeted medications were presented (Fig. 1). A similar protocol design was previously proposed by Vanstraelen et al. (Vanstraelen et al., 2013).

#### 2.3.3 Education and promotion campaign

Educational sessions were held at the hospital during medical and surgical grand rounds attended by all healthcare practitioners prior to the implementation of the protocol. During the presentation, we provided key information about the switch protocol background, rationale, types of medications eligible for switching to PO with examples to each and discussed the benefits from switching to PO therapy. Further, we stressed on the importance of effective discussion and collaboration between healthcare professionals for the successful implementation of the program. Besides, specific education and training was provided to pharmacists and pharmacy interns who worked under the supervision of pharmacists for the execution of the switch process.

#### 2.3.4 Effective implementation of the protocol

The protocol was implemented in April 2018, supported by an implementation campaign where the protocol was distributed inside the pharmacy and at the medication room of the participating wards. Similar support strategies were used by Rawlins & Cerbe (Rawlins and Cerbe, 2006).

Effective implementation was carried out following a 3-step process by prospective identification of all prescriptions that are eligible for IV-PO switch intervention, using a 3-level eligibility assessment (patient eligibility, order eligibility, and clinical eligibility).

**Fig. 2:**
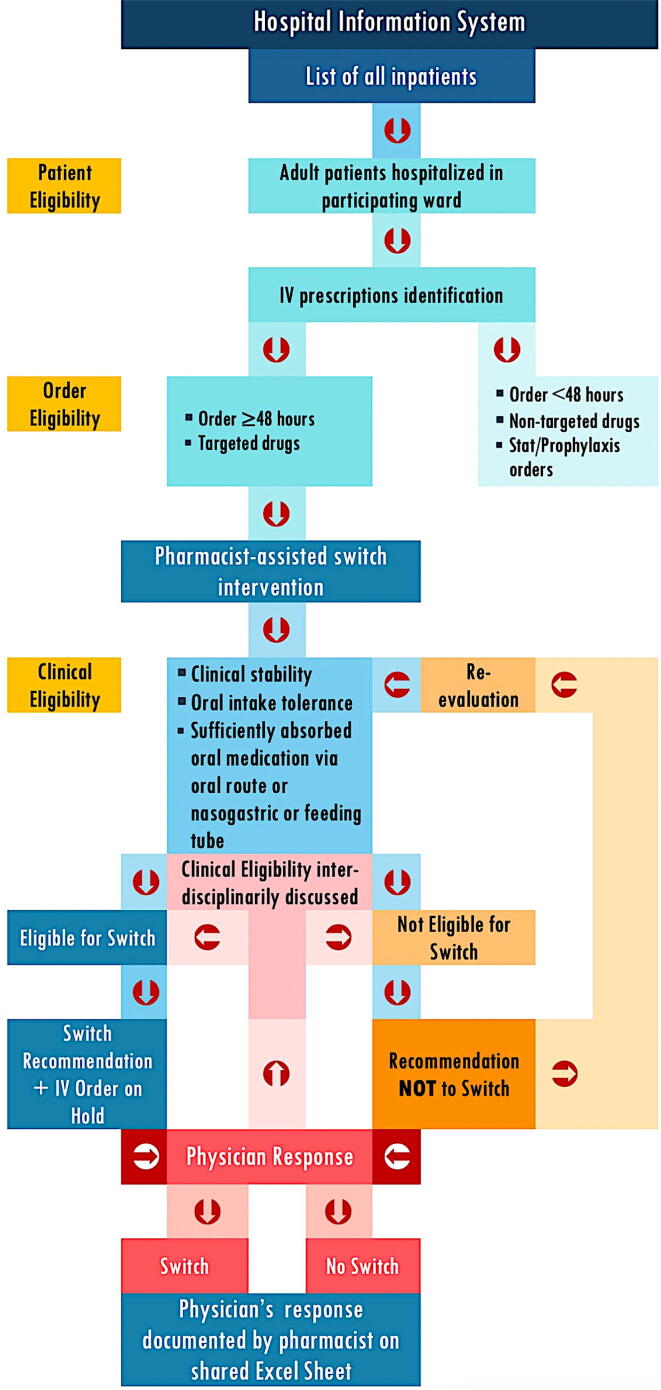
Workflow intervention.

Workflow (Fig. 2) including prospective identification of all adult inpatients in medical and surgical wards (*patient eligibility*), for whom an IV therapy was prescribed for 48 h or more per hospital protocol for switching from IV-PO therapy (*order eligibility*), and who had no clinical condition interfering with PO (*clinical eligibility*). Patient eligibility applied for initially defined target population. Order eligibility applied for targeted drugs ordered for 48 h or more, and excluded IV orders in less than 48 h, STAT, prophylaxis, or prescriptions pertaining to non-targeted drugs. Clinical eligibility, which was done by the pharmacist, applied for clinically stable patients who have adequate absorption of oral medication via oral route or nasogastric tube (NGT), and excluded clinically unstable patients, who were intolerant to oral route, and medications with inadequate absorption due to NGT. Patients whose diagnosis required IV therapy according to specialist guidelines were also excluded (Mertz et al., 2009, Mok et al., 2016, Shrayteh et al., 2014, van den Bosch et al., 2016).

The process results in daily identification of all IV orders that are eligible for PMES. When switch eligibility is identified, the pharmacist flags the concerned IV order through the hospital information system, which automatically sends an electronic message to the prescribing physician and keeps the order on hold, pending physician’s response. Afterwards, the physician’s response (switch or non-switch) is documented side-by-side to the pharmacist’s intervention on a shared Excel sheet, which is updated on a daily basis. In case if physician’s response does not comply with the switch recommendation, the pharmacist contacts the physician to ask for the motivation of switch refusal, and the motivation is documented. If refusal to switch is not motivated, the pharmacist iterates the intervention systematically on a daily basis until compliance. In any case, the final decision to switch is left to the treating physician.

However, if the IV order is deemed not eligible for switch by the pharmacist, the pharmacist’s intervention is documented as “not switch” and paired with the corresponding physician’s action (switch or non-switch).

The physician’s response constitutes the primary outcome of the intervention efficacy and was broadly categorized as “switch” and “no switch”. Switch response was further divided into “switch per hospital protocol” and “switch to another PO alternative”, depending on whether the target PO drug prescribed complies with the promoted hospital switch protocol. Two additional patterns of physician’s response were considered as switch sub-categories, including switch carried out spontaneously by the physician (prior to pharmacist’s intervention) and IV treatment discontinuation (with no PO switch).

“No switch” responses by the physician were classified into “motivated” and “non-motivated” sub-categories. Two principal patterns of “motivated no switch” responses were identified including “PO alternative out of stock”, and “communication issue” pertaining to disrupted pharmacist-physician communication impeding the transfer of switch recommendation which was manifested as inability to send the intervention through the healthcare system for some IV orders and inability to reach the treating physician via phone.

### 2.4 Data collection and outcome definition

Study data was collected for two periods of comparable duration, namely: control phase (November 2017 - March 2018) and intervention phase (April 2018 - August 2018), with an additional follow up phase of 3 months (September 2018 – December 2018) notably to collect readmission data. Two types of data were collected depending on the outcome, as following:

#### 2.4.1 Determination of measurable outcomes

Comparative data was used to analyze safety and cost effectiveness of the intervention and was conducted in control versus intervention phase.

##### 2.4.1.1 Determination of measurable outcomes of safety

Safety analysis was carried out on patients included during a randomly sampled period of one month (4 consecutive weeks) out of the 5 months from each of the two study phases (control vs intervention phase), namely the control and intervention phase. Collected data comprised patients’ demographic, and clinical data including gender, age, diet (no specific diet, NGT, and nil per os route [NPO]), wards (medical, surgical), comorbidities (diabetes, hypertension, etc.), LOS, readmission within 90 days of discharge (yes or no), and in-hospital mortality. These data were collected prospectively from patient’s records. Safety was indicated by readmission rate, LOS, and in-hospital mortality rate.

##### 2.4.1.2 Determination of cost effectiveness

Cost effectiveness analysis data included the overall consumption of IV and PO formulations of targeted medications in the pre- versus post-intervention phases. These data were collected from the pharmacy information system. The respective costs were calculated for each drug separately, based on the purchase prices shared by the Material Management Department of the hospital, and by considering the prescribed dose and frequency of medication. Only prices of the IV or oral drugs were included; costs associated with preparations, administration, pump rental fees & laboratory monitoring were not included. Thus, cost effectiveness outcomes included change in IV consumption and the relative cost saving.

##### 2.4.1.3 Determination of intervention efficacy

Data for intervention efficacy consisted of the pharmacist’s intervention and physician’s response. These data were collected prospectively from the daily intervention follow up excel sheets and were used to estimate the efficacy of the intervention.

The efficacy of the intervention was estimated as the percentage of switch recommendations that resulted in effective switch to oral medication. It was calculated using two different criteria including Direct Efficacy (DE, Criteria A) and Overall Efficacy (OE, Criteria B).

##### 2.4.1.4 Direct efficacy

Criteria A define the efficacy with respect to the direct effect of the pharmacy switch recommendation on modifying the order from IV to PO route. Consequently, DE was estimated using the following formula:

$$\mathrm{DE}\left(\%\right)=100\times \left(\frac{\mathrm{No}.\;of\;modifying\;order+No.\;Discontinuations}{\mathrm{Switch}\;recommendations-Excluded\;according\;to\;Criteria\;A}\right)$$

Exclusion criteria A apply for switches made spontaneously by the physician, motivated negative response by the physician (due to absence of oral alternative or disrupted pharmacy-physician communication), and non-measurable outcome due to patient death or transfer.

##### 2.4.1.5 Overall efficacy

Criteria B assume the effect of overall intervention including direct effect of switch recommendations combined with the effect of education and promotion campaigns that were conducted prior. Thus, IV-PO switch operations that were spontaneously made by the physician was assumed to be the effect of educational intervention; and on the other hand, failure to switch by the physician, in case of disrupted pharmacy-physician communication, reflected inefficacy of the educational intervention. Consequently, OE was estimated using the following formula:

$$\mathrm{OE}\left(\%\right)=100\times \left(\frac{\mathrm{Total}\;no.\;switches+No.\;Discontinuations}{\mathrm{Switch}\;recommendations-Excluded\;according\;to\;Criteria\;B}\right)$$

Exclusion criteria B apply for negative physician’s responses (no switch) that are justified by the absence of oral alternative and non-measurable outcome due to patient death or transfer.

### 2.5 Statistical methods

Data was collected and coded in Microsoft Excel (Microsoft Corporation, 2018) and statistical analysis was performed with the Statistical Package for Social Sciences version 21.0 for Windows (SPSS Inc., Chicago, IL, USA). Categorical variables are presented as frequency and percentage, while discrete variables are presented as mean ± standard deviation (SD) or median and 75th centile (P75) depending on the normality of the distribution. Efficacy of the intervention was estimated as described previously, and the effect of iterative pharmacist interventions was estimated by calculating the number of additional physician’s positive switch responses following second, third and fourth + iterations for the same order with reference to single intervention. Cost effectiveness used cost minimization analysis to compare the difference in overall consumption of both IV and PO medications between control and intervention phases and the associated costs; results are presented as overall consumption and overall costs for each drug with control-to-intervention phase differential indicating the cost savings. Comparison between pre- and post-intervention phase of demographic and clinical data as well as LOS, mortality and readmission rates were carried out using chi-square test for categorical variables, independent *t*-test for normally distributed discrete variables and Mann-Whitney *U* test for non-normally distributed discrete variables. A p-value of < 0.05 was considered to reject the null hypothesis.

## 3 Results

### 3.1 Characteristics of patients in the intervention phase

Of total 1894 patients hospitalized in the participating departments during the 5-month intervention phase, 781 (41.2%) had at least one IV order that met the eligibility criteria. Of these 781 patients, 55.8% were female and 58.7% were hospitalized in surgical wards (male or female). While majority patients had no specific diet (70.4%), 5.0% had NGT and 24.6% had a restriction for oral route for various reasons such as impaired consciousness, specific clinical restriction context such as sepsis, etc. The most frequently prescribed IV medications included gastric acid-reducing agents (GARAs, 50.4%), followed by antibiotics (39.6%), acetaminophen (38.2%), and antiemetics (33.0%); while IV diuretics (furosemide) were prescribed for 15.0% of the patients. The most frequently prescribed IV antibiotics included metronidazole (5.0%), cefazolin (4.9%), and ciprofloxacin (4.6%). A patient may have had more than one IV antibiotic prescription (Table 1).

**Table 1:** Characteristics of patients included in the intervention phase (N = 781).

❖ A patient may have more than one class prescribed in IV route, and more than one medication in the same class. GARAs: Gastric acid-reducing agents.

### 3.2 General characteristics of overall pharmacists’ interventions

Pharmacists carried out a total 2677 interventions, including primary and iterative ones, subsequent to which switch, and no-switch recommendations were emitted in 1185 (44.3%) and 1234 (46.1%) cases, respectively; while no action was made for STAT orders accounting for the 258 (9.6%) remaining cases. The 1185 overall switch recommendations led to favorable physician’s response with effective switches in 277 (23.4% of the 1185) and IV treatment discontinuations in 380 (32.1%); while a negative response was recorded in 488 (41.2%) cases. Consequently, the physician-pharmacist agreement rate regarding switch recommendation can be estimated as 53.0%, after exclusion of transferred and deceased patients.

**Fig. 3:**
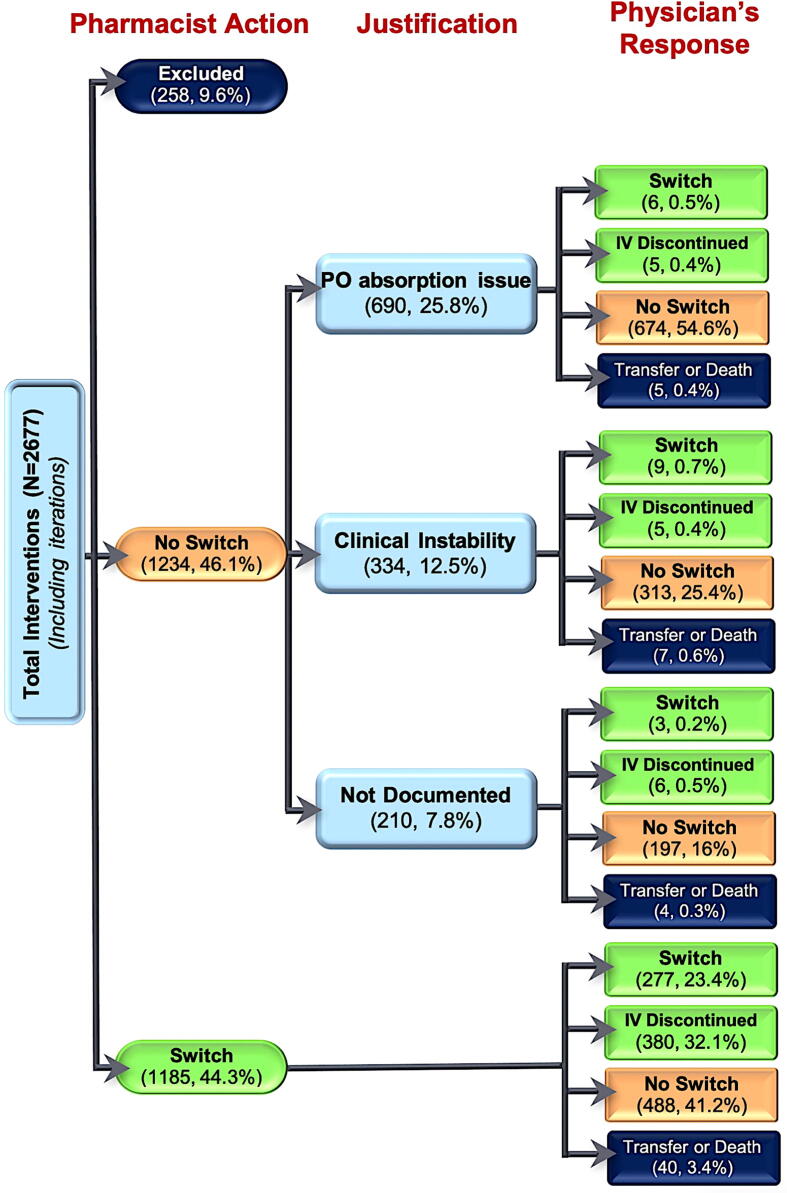
STROBE flowchart of overall pharmacist interventions and their outcomes.

On the other hand, the 1234 pharmacist’s no-switch recommendations were justified by PO absorption issue in 690 cases (25.8%), clinical instability in 334 cases (12.5%), and no justification was mentioned in the remaining 210 (7.8%) cases. The physician’s decision was in agreement with no switch recommendation in majority cases, except in 34 cases where the treatment was switched to PO form despite the pharmacist’s unfavorable recommendation (physician-pharmacist agreement rate = 97.2%, after exclusion of the transferred and deceased patients). Fig. 3 presents the flowchart of all pharmacy interventions including pharmacist recommendation for switch or no switch, justification for no switch, and physician’s response (switch, discontinuation, no switch or patient death or transfer).

### 3.3 Outcomes of the switch recommendations from primary pharmacy intervention

By exclusion of iterative interventions (reinterventions for the same orders), switch recommendations from primary interventions (N = 677) led to effective switch in 60.7% cases. The reasons for effective switch included switch to the same PO medication recommended by the pharmacist (8.9%), switch to another PO medication (2.5%), spontaneous switch by physician (17.6%) and IV order discontinuation (31.8%). On the other hand, absence of effective switch accounted for 36.9% of primary switch decisions. The reasons included absence of PO alternative in 7 (1.0%) and communication issue in 43 (6.4%) of the cases; whereas IV treatment was continued in 28.4% or switched to another IV medication in 1.2%. Based on these findings, efficacy of the pharmacy intervention related to the Direct Efficacy of the primary switch recommendation was estimated as 59.3%, while Overall Efficacy was estimated as 62.8% (Table 2).

**Table 2:** Outcome of primary pharmacist’s switch recommendations (N = 677) and estimation of the intervention efficacy.

**Criteria A**: define the efficacy with respect of the direct effect of the pharmacy switch recommendation on modifying the order from IV-PO route.

**Criteria B**: assume the effect of the overall intervention including the switch recommendation combined with the priorly conducted educational intervention, thereby assuming that IV-PO switch operations that were spontaneously made by physician are the effect of the educational intervention and, on the other hand, failure to switch by the physician, in case of disrupted pharmacy-physician communication, reflect inefficacy of the educational intervention.

### 3.4 Estimated effect of iterative pharmacist interventions on physician’s response

**Fig. 4:**
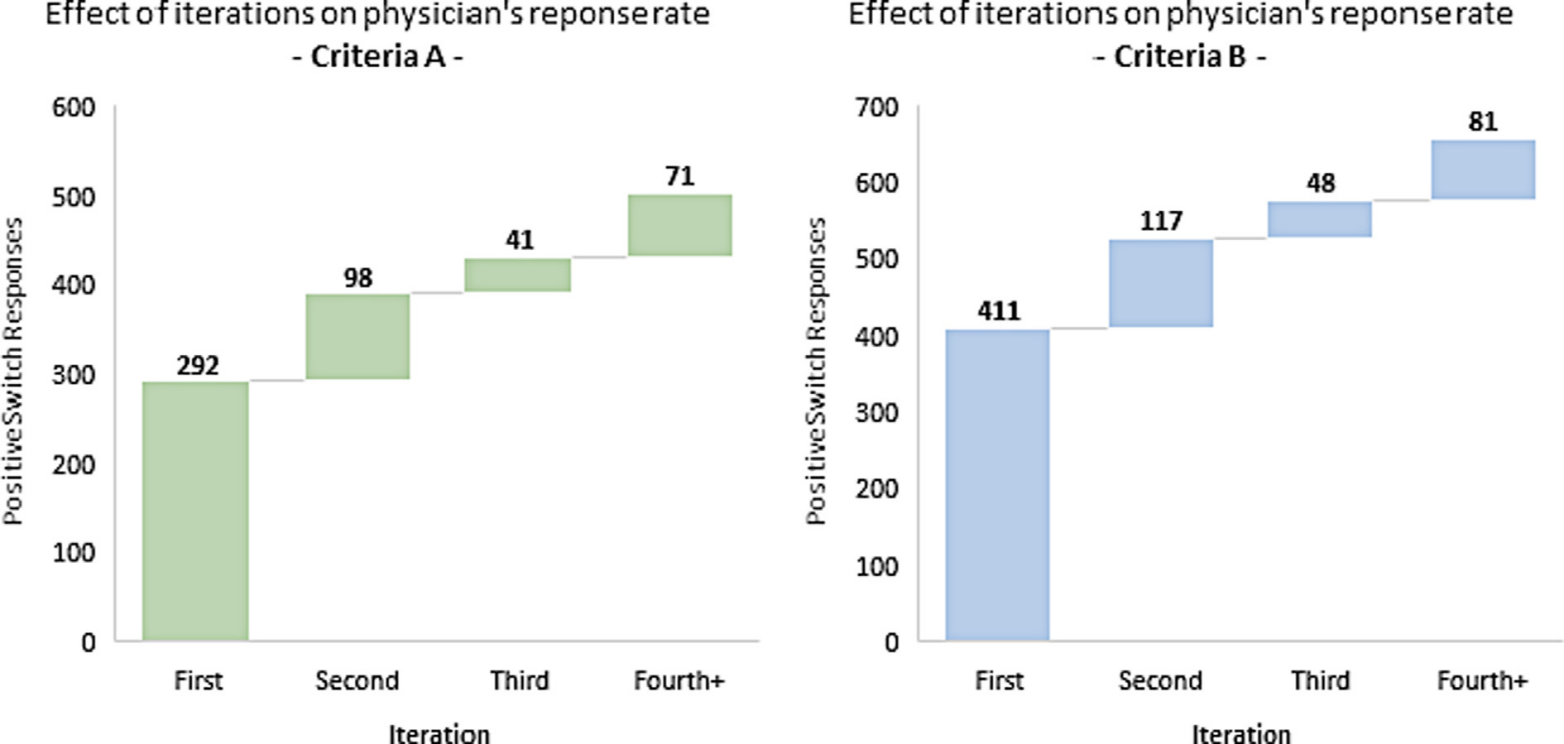
Estimated effect of iterative pharmacist interventions on physician’s response.

With respect to efficacy criteria A, the second, third, fourth and further iterations resulted in additional 98, 41 and 71 IV-PO switches, respectively. With respect to efficacy criteria B, the second, third, fourth and further iterations resulted in additional 117, 48 and 81 IV-PO switches, respectively (Fig. 4).

### 3.5 Effect of the intervention on IV and PO medication consumption

**Fig. 5:**
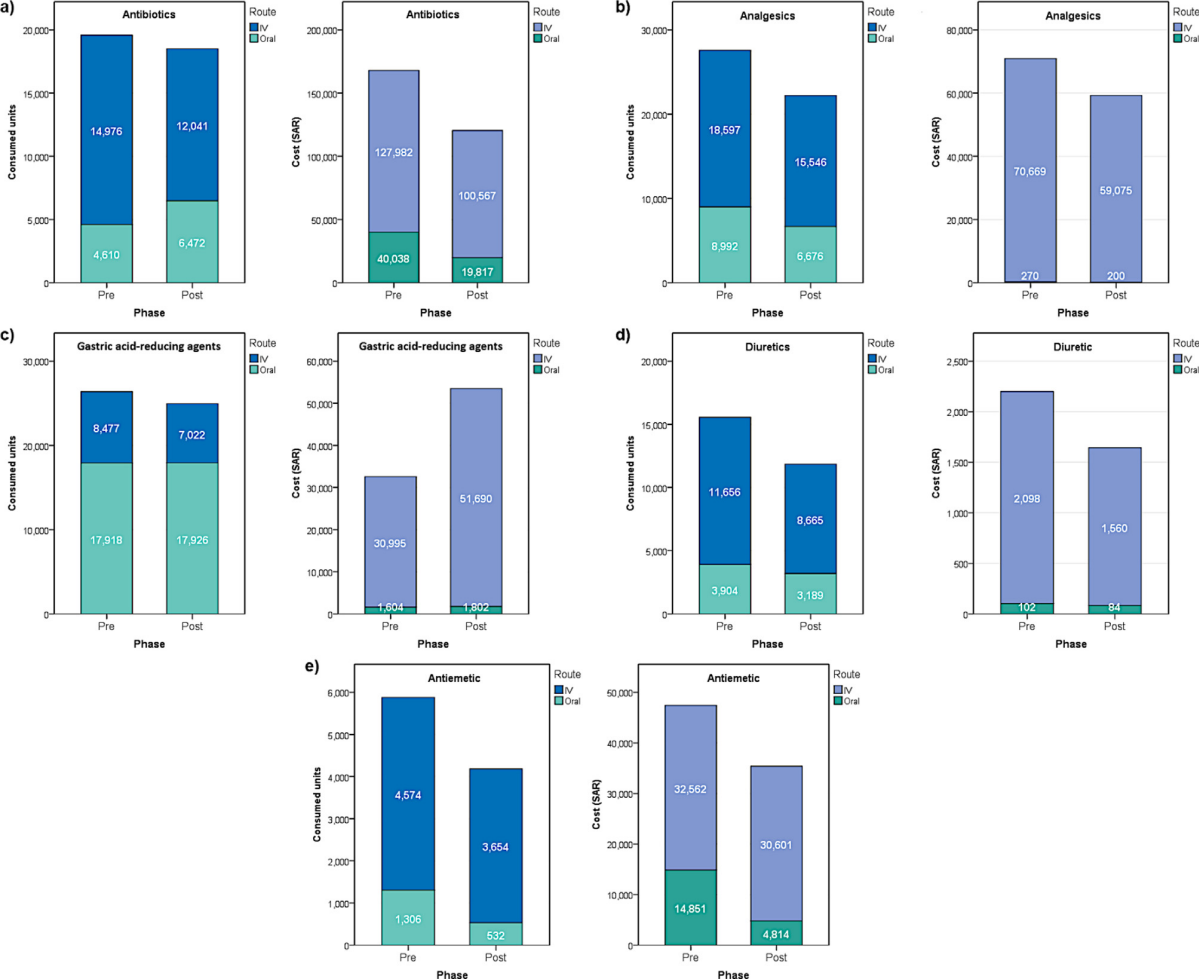
Effect of the intervention on IV and PO medication consumption and cost.

The intervention was associated with reduced IV medication consumption from 4,574–18,597 vials in control phase to 3,654–15,546 vials in post phase, depending on the therapeutic class. Surprisingly, we also observed reduction in oral medication consumption concerning all therapeutic classes except antibiotics, where oral consumption increased from 4,610 to 6,472 tablets in control to intervention phase, respectively (Fig. 5). However, by reference to overall consumption, the proportion of oral medications has increased from 38.7% in pre- to 57.5% in post-intervention, while IV has declined. This increase in proportional use of oral forms was more remarkable in antibiotics (from 23.5% to 35.0%) and GARAs (from 67.9% to 71.9%) (Results are not presented in Tables or Figures).

### 3.6 Cost effectiveness analysis

The decrease in IV and PO consumption was associated with significant decrease in medication cost for all therapeutic classes, except for GARAs. This might be due to increased prescription of pantoprazole 40 mg IV (from 965 vials to 3,028 vials) which is costly (14,06 SAR per vials) compared to omeprazole 40 mg IV (2,63 SAR) (Fig. 5).

**Fig. 6:**
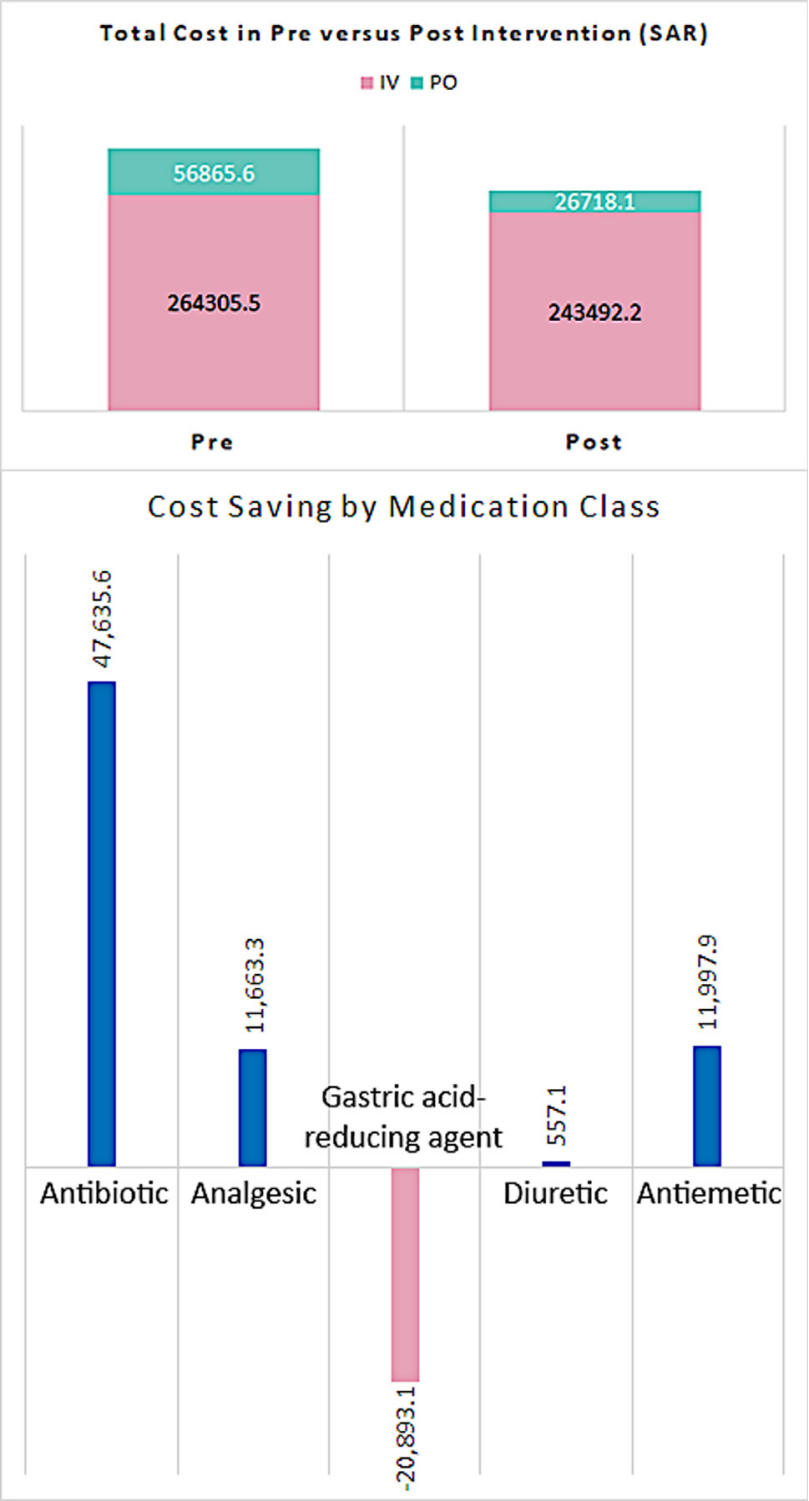
Overall and by medication class pre to post-intervention cost saving.

Consequently, the intervention enabled overall cost saving of 50,960.8 SAR ($13,589.5), which represents an average monthly cost saving of 10,192.2 SAR ($2,717.9). The most significant cost saving was made in antibiotics accounting for 47,635.6 SAR ($12,702.8) while a marginal cost saving of 557.1 SAR ($148.6) was made in diuretics and a loss of 20.893.1 SAR ($5,571.5) was observed in GARAs (Fig. 6).

### 3.7 Safety of the intervention

The safety outcome of the interventions was assessed in randomly sampled 158 patients and 137 patients in the control and intervention phases, respectively (Table 3). These showed relative reduction in mean (26.74 to 19.34 days, p = 0.012) and median (14.5 versus 12.0 days, p = 0.155) hospital LOS, without significant increase in readmission rate (47.5% versus 57.7%, p = 0.080) and no effect on mortality (22.2% versus 19.7%, p = 0.607) in control versus intervention phase, respectively. Further, no differences in gender, age, diet and diagnosis were observed between the two samples.

**Table 3:** Assessment of the intervention’s safety including mortality and length of stay.

*Statistically significant result (p < 0.05).

## 4 Discussion

### 4.1 Summary of findings

During intervention phase, we identified 677 IV orders that were eligible for switch among 781 patients, accounting for nearly 9 inappropriate IV prescriptions in every 10 patients, on an average. A total 1185 PMES IV-PO switch interventions were carried out, resulting in an overall 55.5% response rate. However, according to the efficacy model developed in this study, the 677 primary interventions lead to 62.8% efficacy rate, after exclusion of cases where the oral alternative was out of stock as well as transferred and deceased patients. On the other hand, repeat interventions and reminders (up to 4 and more repeat interventions) were necessary on the remaining switch-eligible prescriptions, and enabled 246 additional IV-PO switches. The efficacy model further enabled the calculation of the individual effect of the educational program, which was estimated as 3.5% of the 62.8% efficacy rate, while the remaining 59.3% was attributed to the stewardship program. Indicators’ analysis showed up to 3,050 vials reduction in IV medication consumption, depending on the therapeutic class, and an overall cost saving as high as 50,960.8 SAR ($13,589.5), 93.5% of which was made on antibiotics. Safety analysis showed one-week reduction in mean hospital LOS from pre- to post-intervention time, with no significant increase in readmission or mortality rates.

### 4.2 Assessment of switch eligibility and initial intervention

One of the challenging actions of this program is to adequately assess eligibility for switch of IV prescriptions. The difficulty was enhanced by the relatively high percentage of patients on tube feeding or with NPO route (29.6%). These cases not only required accurate assessment of the practicability of oral route, but also necessitated daily monitoring of their status in case of temporary restriction. Accurate assessment of appropriateness of IV-PO medication switch may be difficult even to clinicians, and in many cases the decision stands on the physicians’ clinical intuition, which may conflict with the evidence based recommendations and objective criteria that constitute the basis of the pharmacists’ action (Iversen et al., 2019, Lee et al., 2012, Mok et al., 2016, Shrayteh et al., 2014). Therefore, interdisciplinary decisions for switch may be necessary for some patients after exposing bioavailability of the available oral alternatives and discussing the practicability and eventual benefits of the oral route with respect to the patient’s condition. This emphasizes the role of communication and inter-professional education in improving doctor-pharmacist relationship for enhanced patient’s care (Gallagher and Gallagher, 2012). Nevertheless, the very high physician-pharmacist agreement rate (97.2%) regarding no switch recommendation, in the present study, may be a reliable reflection of the pharmacist’s accuracy in the clinical assessment of the patient. The same deduction cannot be made with regards to switch recommendations, which yielded lower agreement rate (53.0%), as switch eligibility was assessed at both the IV order and patient’s clinical data levels, using both the objective criteria from the hospital protocol and interdisciplinary discussion over the iterative interventions for non-switched prescriptions. Consequently, only 2.8% of the negative physician’s response to switch was motivated, as per Criteria B for the intervention efficacy, justified by the unavailability of the oral alternative in the hospital. This leads us to conclude that the disagreement regarding the switch recommendations is mainly due to physicians’ non-adherence with the protocol and not to clinically justified decision. Hence, findings based on this model can be considered accurate and reliable.

### 4.3 Role of education and its contribution in the efficacy of the intervention

The education of healthcare staff in the principles of switch therapy and the distribution of appropriate guidelines is essential for a successful IV-PO switch program (Rawlins and Cerbe, 2006). In our institution, the educational campaign that preceded the protocol implementation included majority of the medical staff. Besides, presenting the switch protocol and the benefits for the patient and the community of such commitment, we stressed the importance of effective discussion and collaboration between healthcare professionals for the successful implementation of the program similar to previous studies (Ho et al., 2005, McLaughlin et al., 2005). Additionally, specific education and training for the execution of the switch process were provided to the pharmacists and pharmacy interns who worked under supervision of pharmacists. However, the efficacy of education alone may be debated.

Although the present model estimating the individual efficacy of the educational intervention as the difference in efficacy between Criteria A and Criteria B may be mathematically valid, it remains difficult to discriminate the contribution of the educational intervention in the overall program efficacy. Indeed, acceptance and adherence to the switch recommendation during the intervention phase was probably enhanced by prior exposure of the physicians to the awareness and education campaign. Thus, the efficacy of physician’s education to early IV-PO switch is probably underestimated by the present model. Previous evidence showed considerable effect of purely educational programs in improving the percentage of switched prescriptions; however, the effect of such intervention was not sustainable, and the efficacy declined over time (van Niekerk et al., 2012).

### 4.4 Relevance of combined interventions

Generally, observations from studies using single method showed relatively lower efficacy than combined methods. This was the case of Mertz et al., who evaluated the efficacy of implemented check list with criteria for IV-PO switch in a selection of antimicrobials, without any further inductive action. The results showed a reduction from 6 to 5 of the median number of days of IV treatment by patient (Mertz et al., 2009). Another example is the computer-generated reminders for switch used by Fischer et al., which showed only 11% decline in average IV daily dose by admitted patient and only 35% of positive physician’s response rate, while total drug expenditure increased by 12% by comparison to the control period (Fischer et al., 2003). Likewise, a study by Hammad et al. assessed the effectiveness of implementing criteria checklist for IV-PO switch among physicians. This reported 36% of switch rate among patients on IV medication who were eligible for oral route, (Hammad et al., 2015).

By contrast, Rawlins & Cerbe reported high switch acceptance rates (85% and 87%) along with significant increase in the appropriate switch timing (from baseline 17% to 88% and 90% of the prescriptions) subsequent to pharmacist intervention alone and with the contribution of an Infectious Disease Physician respectively, following an educational program to the medical, pharmacy and nursing staff (Rawlins and Cerbe, 2006). On the other hand, a phone call-based pharmacist intervention alone, with daily monitoring of the physician’s response, resulted in nearly 60% benefiting from immediate switch, while refusal to switch was clinically motivated in 73% of the remainders (Vanstraelen et al., 2013). These levels of efficacy are comparable to our findings showing initial switch response rate of 62.8%, which was considerably increased after repeat interventions, accounting for 246 additional switch actions. However, the estimation of the ultimate efficacy rate could not be calculated as the relative cumulative number of switch eligible prescriptions has changed over the follow up days while the related variable was not collected.

These observations underscore the pertinence of combining education with direct pharmacy intervention to solicit the oral switch and monitor the physician’s response for each eligible IV prescription while eliciting inter-professional communication, thereby maximizing the effect of the intervention. We have previously demonstrated the efficacy of such combined strategy in improving antimicrobial prescribing practice in our institution, with a maintained effect (Alawi and Darwesh, 2016).

### 4.5 Cost effectiveness

The direct measurable indicator of the switch intervention effectiveness is the reduction in IV medication consumption and the related cost saving, as adjusted to the expected increase in PO medication consumption. However, findings from the present study showed decrease in both IV and PO medication consumption, except for antibiotics where consumption of PO forms increased as expected. Reduction in PO consumption was an unexpected outcome, which could be indirectly related to the awareness raising effect of the intervention, where physicians feel the need to regularly revise the prescriptions and evaluate the usefulness of each medication including oral ones. This was associated with an overall cost saving of 50,960.8 SAR ($13,589.5) over 5 months.

Although the amount of raw cost savings is not comparable across different studies, due to differences in medication prices, patients flow, intervention duration and use of different calculation methods, the health economics dimension of such interventions is often highlighted. A comparable combined-method intervention, which was implemented stepwise (over 5 years) in a French hospital, enabled an average 17% reduction in IV-PO prescribing ratio and 10% decrease in vials cost of all drugs prescribed inpatient. This resulted in an estimated cost saving of approximately $2.6 million in the last year of the study when compared to the baseline (Corny et al., 2017). Another study from Johns Hopkins Hospital focused on expensive IV medications such as chlorothiazide, voriconazole, levetiracetam, and pantoprazole estimated yearly cost saving of approximately, $1.2 million if bioequivalents PO forms were prescribed to the eligible patients (Lau et al., 2011). These observations underscore lack of awareness among physicians about the drugs costs, which leads to the prescribing decision being totally independent of the drug price. This is supported by observation from our study showing increase in proton pump inhibitors related costs in the intervention phase despite a decrease in the IV consumption. By further analyzing this paradoxical finding, we found that this was due to unexplained shift in IV prescribing preference from ranitidine ($0.17 per unit) in control phase to pantoprazole ($3.75 per unit) during the intervention phase, which increased the costs in this treatment category.

By far, the major share of cost savings in the present study was attributed to antibiotics, calculated as 47,635.6 SAR ($12,702.8), over 5 months, which represented 93.5% of the total cost savings. Antibiotics constitute the largest proportion of drug expenditure in inpatient settings, of which IV forms account for nearly one-third (Mandell et al., 1995). Reports from America estimated between $2.5–3.5 billion the nationwide yearly expenditure on antibiotics in inpatient settings only (Suda et al., 2018, Suda et al., 2013). Nonetheless, the issue of inpatient antimicrobial expenditure should be viewed in the wider scope of inappropriate universal use, and not only inappropriate IV prescribing. This highlights the necessity to conduct antimicrobial stewardship programs to improve antibiotic prescribing practice and reduce both qualitative and quantitative misuse (Alawi and Darwesh, 2016, Doron et al., 2013). Of note, although consumption of GARAs decreased, we observed an increase in the associated costs. This is explained by omeprazole being out of stock in the second phase (intervention phase), resulting in selective use of pantoprazole, which is over 5 times more expensive than omeprazole.

### 4.6 Safety

Several studies showed comparable effectiveness and safety of various PO medications use instead of their IV bioequivalent in different conditions, including severe conditions such as peptic ulcer with bleeding (Tsoi et al., 2013), infectious endocarditis (Iversen et al., 2019), and febrile neutropenia in cancer patients (Vidal et al., 2013). Additionally, good safety outcomes are consistently reported from IV-PO switch interventions. These demonstrated reduction in time to clinical stability and hospital LOS with no relapse of the treated condition or increase in readmission or mortality rates (Iversen et al., 2019, Mertz et al., 2009, van Niekerk et al., 2012). Similar to these findings, we reported a significant reduction of LOS, with mean duration reduced by 7 days and median by 2 days, approximately; whereas readmission and mortality rates were comparable in the two phases. Besides being a safety outcome, owing to the increased risk of nosocomial diseases and other hospitalization related health risk, the reduction in LOS could be included in the estimation of the saving on health expenditure by the patient. Further, these safety outcomes are to be combined to the risk reduction in IV complications including IV line associated thrombophlebitis and infections.

## 5 Limitations

There are some methodological limitations in this study that need to be taken into account for future consideration. Further, medication consumption was accounted for the dispensed units from the pharmacy system (i.e. vials and tablets) in both study phases, and by consequence the returned or unused medications units where not deducted. Finally, and most importantly, the data collection sheet was designed as intervention based instead of prescription-based observations. This means that each observation corresponded to the pharmacist intervention; this made the analysis challenging notably to follow up the prescriptions over the hospitalization stay and to discriminate repeat interventions from patient’s readmission. However, this issue did not interfere with safety and cost effectiveness analysis as their data was collected separately. Unfortunately, this study does not measure true knowledge of doctors. Future idea is to retrieve doctor’s feedback through intervention screen.

## 6 Conclusion

Combined system enhancements, educational presentations and pharmacist assisted protocol implementation to prompt early IV-PO switch in inpatient setting are effective, cost-effective and safe methods. These interventions should be encouraged and evaluated in all healthcare institutions and be part of a strategic health quality and economic vision, both at the governmental and institutional levels. Beyond harmonizing of the prescribing practice and levelling with the evidence-based recommendations, such programs contribute in inter-professional education and communication and enable multidisciplinary focus on the patient’s unique case while considering the underlying public health issues. We stress that both physicians’ education and pharmacist intervention are essential to achieve the best efficacy. While the present intervention was carried out on the only initiative of the institution’s pharmacy staff and designed for the institution, local health authorities should consider establishing national standard methods in a comprehensive strategy to improve medication use and fight against misuse.

## Declaration of Competing Interest

The authors declare that they have no known competing financial interests or personal relationships that could have appeared to influence the work reported in this paper.
